# Validation of the Greek Translation of the Nursing Dimensions Inventory questionnaire (NDI-35)

**DOI:** 10.5539/gjhs.v6n5p30

**Published:** 2014-05-08

**Authors:** Evagelia Kotrotsiou, Mary Gouva, Stiliani Kotrotsiou, Maria Malliarou, Theodosios Paralikas

**Affiliations:** 1 Head School of Health and Welfare Professions T.E.I. of Thessaly, Greece; 2 Higher Technological Educational Institution of Epirus, Greece; 3 Nursing Department T.E.I. of Thessaly, Greece; 4 Nursing Department T.E.I. of Larissa, Greece

**Keywords:** nurses, nursing, care, NDI-35 questionnaire, validation, reliability, validity

## Abstract

**Context:**

The concept of care is a fundamental issue in nursing science. Therefore the development and the use of tools for assessing care is an imperative for the nursing profession. The NDI-35 questionnaire is one such tool for assessing the nursing care.

**Objectives:**

The purpose of this paper is to adapt and use the NDI-35 questionnaire in Greek nursing practice. A translation and validation of NDI-35 questionnaire is performed.

**Methods:**

Exploratory factor analyses, as well as internal consistency and test–retest analyses, were conducted.

Forward translations from English were produced by three independent Greek translators and then back translations by five independent bilingual translators. The Greek NDI-35 questionnaire that was produced was administered to 200 nurses (144 women and 56 men) from tertiary and secondary health care facilities. Data were analyzed using principal component analysis and Cronbach’s alpha.

**Results:**

One hundred and eighty four nurses that answered the NDI-35 questionnaire were graduates from the Technological Educational Institute (T.E.I.) and 64% of the respondents had more than 15 years of professional experience. Two subscales arbitrarily called “clinical work” and “patient needs” emerged, with the mean “clinical work” subscale score being at 70.16 ±12.90 (a maximum of 85) and mean “patient needs” subscale at 21.49± 6.16. Considerable differences in scoring among different items were observed when the NDI-35 answers were compared to their Greek counterparts’. Results confirmed that: (a) the translated versions are an accurate translation of the original, (b) factor analyses established similar factor solutions as that of the English versions, (c) reliability coefficients are satisfactory (i.e., Cronbach’s α coefficients and test–retests), and (d) construct validity revealed similarities between English and Greek versions, replications consistent with past research, as well as differences explained through theoretical frameworks. Therefore, both scales were accepted as valid and reliable measures in Greek-speaking populations.

**Conclusion:**

Alphas and test-retest correlation suggest the Greek translated and validated NDI-35 questionnaire is a reliable tool for assessing nursing care. Factor analysis and focus group input suggest it is a valid tool. Nurses in different settings may perceive nursing care differently. The findings of the current paper are discussed in the context of nurse education and assessment of care.

## 1. Introduction

Care is considered a fundamental concept for human beings (Heidegger, 1975). It consists the “human way” of existence in every relationship and it goes beyond the simple “sympathy” (Mayeroff, 1971). However, care, as a concept, as many other abstract concepts, such as beauty, kindness, and love still remains elusive. Caring and nursing have always been thought of synonymously (Vance, 2009). There are many definitions of nursing, such as holism, caring, teaching, advocacy, supporting, promoting, maintaining and restoring health are all components of nursing practice (Akansel, Watson, Aydin, & Ozdemir, 2011).

Caring is a complex, essential and crucial component of nursing (Roach, 1991; Schoenhofer, 2001). Caring is an expression of humanity as Watson reaffirmed by stating that “… caring is a moral ideal, rather than an interpersonal technique and it entails a commitment to a particular end. The end is protection, enhancement, and preservation of the person’s humanity… “(Watson, 1988). Caring is not an easy concept to measure (Beck, 1999). Various researchers have attempted to quantify the phenomenon of care and they resulted in developing many different instruments (Wolf, 1986; Watson & Lea, 1997; Hsieh, Kuo, Tseng, & Turton, 2005). Perceptions of nurses about nursing care and patients’ expectations from nursing care are different as it is shown in a variety of studies. McCance et al.’s study (2009) has shown that the perception of the nurses and patients with regard to nursing care were found to be congruent on statements related to technical and intimacy aspects of nursing.

Different instruments to measure care exist such as Care-Q instrument developed by Larson (1984), the Caring Behaviors Inventory (CBI) (Moyle, Iselin, Baeslack-Smith, & Fleming, 2005), the Caring Behavior Assessment (CBA) (O’Connell & Landers, 2008) and the Caring Dimensions Inventory (CDI) (McCance, Slater, & McCormack, 2009). Only CBI is translated and used in Greek studies, so a need for Greek nursing literature to be enriched made this research imperative.

Watson and Lea (1997) developed the CDI-25 (Caring Dimensions Inventory-25) by operationalizing 25 distinct aspects of nursing which were verified in the literature. Few years later, the CDI-35 was developed, incorporating more items on patient-nurse interaction and spirituality (Watson, Deary, & Lea, 1999, 2001). Watson et al., (2003) research team further modified the CDI-35 Caring Dimensions Inventory and constructed an updated version, which they termed as the Nursing Dimensions Inventory (the NDI-35). The current study was designed to investigate further the structure of NDI-35 in its Greek version and to assess its factorial validity and its internal consistency. Internal consistency is typically a measure based on the correlations between different items on the same test. It measures whether several items that propose to measure the same general construct produce similar scores. When factorial validity is acceptable, it means each measurement item correlates strongly with the one construct it is related to, while correlating weakly or not significantly with all other constructs (Ouzouni & Nakakis., 2011).

## 2. Material and Methods

The current study was conducted at the University Hospital of Larissa and at the General Hospital of Larissa during spring of 2012. Convenience sampling was used as to collect the data. Registered nurses working in internal medicine and surgery sector compromised the sample. Questionnaires were distributed to a total of 200 nurses who have already given their consent to participate in the study. The Greek and English versions of NDI-35 are shown in Table 1.

**Table 1:** Simultaneous presentation of the English and Greek version of NDI-35

Stem question: ‘As a nurse it is/will be important for me to:’ Response on a 5-point Likert scale: 1 (very important) to 5 (not at all important)	
1.Involve a patient with his or her care	1. Να εμπλέκεις έναν ασθενή στη φροντίδα του/της
2. Give reassurance about a clinical procedure	2. Να καθησυχάζεις τον ασθενή σχετικά με μια κλινική διαδικασία
3. Pray for a patient	3. Να προσεύχεσαι για τον ασθενή
4. Deal with everyone’s problems at once	4. Να ασχολείσαι με τα προβλήματα του κάθε ασθενή ατομικά
5. Observe the effects of a medication on a patient	5. Να παρατηρείς τις επιδράσεις ενός φαρμάκου στον ασθενή
6. Keep in contact with a patient after discharge	6. Να κρατάς επαφή με τον ασθενή μετά την έξοδό του από το νοσοκομείο
7. Assure a terminally ill patient that he or she is not going to die	7. Να διασφαλίζεις σε έναν ασθενή τελικούσταδίου ότι δεν πρόκειται να πεθάνει
8. Stay at work after a shift has finished to complete a job	8. Να παραμένεις στην εργασία σου μετά το τέλος της βάρδιας για την ολοκλήρωση μιας δουλειάς
9. Come to work if I am not feeling well	9. Να έρχεσαι στη δουλειά, όταν δεν αισθάνεσαι καλά
10. Attend to the spiritual needs of a patient	10. Να φροντίζεις τις πνευματικές ανάγκες ενός ασθενή
11. Be cheerful with a patient	11. Να είσαι ευχάριστος με τους ασθενείς
12. Provide privacy for a patient	12.Να προστατεύεις την ιδιωτικότητα του ασθενή
13. Make a patient do something, even if he or she does not want to	13. Να βάζεις έναν ασθενή να κάνει κάτι, έστω και αν αυτός δεν θέλει
14. Appear to be busy at all times	14.Να δείχνεις ότι είσαι απασχολημένος συνέχεια
15. Arrange for a patient to see his or her chaplain	15. Να κανονίζεις να δει τον ασθενή ο εφημέριός του/της
16. Assist a patient with an activity of daily living (washing, dressing, etc.)	16. Να βοηθάς τον ασθενή σε μια δραστηριότητα της καθημερινής ζωής (πλύσιμο, ντύσιμο, κ.λπ.)
17. Keep patient records up to date	17. Να διατηρείς τα αρχεία των ασθενών έως σήμερα
18. Feel sorry for a patient	18. Να αισθάνεσαι λύπη για κάποιον ασθενή
19. Get to know the patient as a person	19. Να αναγνωρίζεις τον ασθενή ως πρόσωπο
20. Explain a clinical procedure to a patient	20. Να εξηγείς μια κλινική διαδικασία στον ασθενή
21. Be neatly dressed when working with a patient	21 Να ντύνεσαι προσεγμένα όταν εργάζεσαι με ασθενείς
22. Sit with a patient	22. Να κάθεσαι με έναν ασθενή
23. Explore a patient’s lifestyle	23. Να διερευνάς τον τρόπο ζωής του ασθενή
24. Report a patient’s condition to a senior nurse	24. Να αναφέρεις την κατάσταση του ασθενή σε έναν ανώτερο ιεραρχικά νοσηλευτή
25. Be with a patient during a clinical procedure	25. Να είσαι δίπλα στον ασθενή κατά τη διάρκεια μιας κλινικής διαδικασίας
26. Be honest with a patient	26. Να είσαι ειλικρινής με τον ασθενή
27. Organize the work of others for a patient	27. Να οργανώνεις την εργασία των άλλων για τον ασθενή
28. Listen to a patient	28. Να ακούς τον ασθενή
29. Consult with the doctor about a patient	29. Να συμβουλεύεσαι τον γιατρό για τον ασθενή
30. Instruct a patient about an aspect of self-care (washing, dressing, etc.)	30. Να καθοδηγείς τον ασθενή για αυτοφροντίδα (πλύσιμο, ντύσιμο, κ.λπ.)
31. Share a personal problem with a patient	31. Να μοιράζεσαι ένα προσωπικό πρόβλημα με κάποιον ασθενή
32. Keep relatives informed about a patient	32. Να κρατάς ενήμερους τους συγγενείς για ότι αφορά τον ασθενή
33. Measure the ‘vital signs’ of a patient (e.g. pulse and blood pressure)	33. Να μετράς τα “ζωτικά σημεία” του ασθενή (π.χ. σφυγμό και αρτηριακή πίεση)
34. Put the needs of a patient first (i.e. before your own)	34. Να βάζεις τις ανάγκες του ασθενή πρώτα (δηλαδή πριν από τις δικές σου)
35. Be technically competent with a clinical procedure	35. Να είσαι τεχνικά επαρκής σε μια κλινική διαδικασία

Instrument

NDI-35 is, essentially, a self-assessment of perceptions of caring. There is a stem question (‘‘do you consider the following aspects of your nursing practice to be caring’’) and for each of the items in the questionnaire (e.g. ‘‘listening to a patient’’; ‘‘measuring the vital signs of a patient’’; ‘‘making a nursing record about a patient’’) the respondent is required to indicate on a 5-point Likert scale ranging from ‘‘strongly agree’’ to ‘‘strongly disagree’’ how they perceive caring. Five key factors were identified in the CDI-35 and these includes Technical, Unnecessary, Supporting, Intimacy and inappropriate and the NDI -35 includes further two factors, namely: Factor 1 was labeled ‘Important aspects of nursing’ and Factor 2 was labeled ‘Unimportant aspects of nursing’ (Watson, Deary, & Lea, 2001).

Translation Procedure

The most commonly applied translation process for questionnaires or inventories is the forward– backward translation (Yu, Lee, & Woo, 2004). The first step of this procedure involves a forward translation from the original language (English) to the language intended to be translated in (Greek). The second step includes back translation from the Greek to the original language (English) and consequently compared to the original version. Inaccuracies in the intended language are simply identified through differences in meaning that occur in the backward translation (Wang, Lee, & Fetzer, 2006).

The result differences were checked by a third scientist who made the necessary adjustments. The final version of the questionnaire was presented to a small group of nurses who confirmed that the Greek version of NDI-35 is coherent and easy to fill in.

## 3. Test-Retest Reliability

Three months later the NDI-35 (shorter version) was re-administered to a randomly chosen subset of the original sample (*N*=50), in order to assess the test-retest reliability. At the retest period for this subset Cronbach’s α, was .85. The test-retest reliability was *r* =.82 (*p*<.001) for the total NDI-35. On a subscale level the correlation was: “clinical work” *r* =.91, “patient needs” *r* =.81.

***Statistical analysis:*** Data were analyzed by exploratory factor analysis using principal components analysis (PCA) followed by Varimax rotation using SPSS for Windows version 17.0. The appropriateness of data was checked with Kaiser – Meyer - Olkin (KMO) measure, which examines correlations among the items and its values should be greater than 0.6 in order for a satisfactory analysis to be achieved. The KMO value in the present study was 0.62

Factor analysis reduces multivariate data to fewer underlying dimensions (Hair, Anderson, & Tatham, 1987). PCA enables as much of the total variation in the data to be explained in as few factors as possible, and also provides a rationale for selecting the number of latent factors present. The number of factors extracted was decided after using the scree slope method of analysis (Child, 1990) as opposed to the eigenvalues greater than one rule which can overestimate the number of factors (Cliff, 1988). In order to characterize factors the rotational procedure was used which maximizes the loading (correlation) of items with their putative factors while minimizing the loadings of these items with the remaining putative factors (Kline, 1994). Putative factors were further analyzed for internal consistency, which is one form of reliability (Polit & Hungler, 1995), using Cronbach’s alpha. The result factors create the respective subscales (arbitrarily called “clinical work” and “patient needs”). As a cut off value for an item to enter the subscale, the value 0.40 was set. Rotated factor loadings and Cronbach α are shown in Table 2.

**Table 2:** Rotated factor loadings

Items	Factors	
	1.Clinical work	2.Patient needs
**1**	**0.453**	0.177
**2**	**0.756**	-0.045
**3**	0.130	0.225
**4**	0.127	**0.751**
**5**	**0.677**	0.196
**6**	-0.329	0.360
**7**	0.100	0.362
**8**	0.262	0.343
**9**	0.074	0.006
**10**	0.120	**0.659**
**11**	**0.860**	0.025
**12**	**0.837**	-0.028
**13**	-0.047	0.073
**14**	-0.390	0.104
**15**	0.013	**0.748**
**16**	**0.657**	0.368
**17**	0.143	0.524
**18**	0.231	0.198
**19**	**0.632**	0.303
**20**	**0.617**	0.185
**21**	**0.816**	0.123
**22**	0.103	**0.739**
**23**	0.014	**0.651**
**24**	**0.502**	0.370
**25**	**0.788**	0.023
**26**	**0.565**	0.070
**27**	0.295	0.199
**28**	**0.876**	0.108
**29**	**0.898**	0.040
**30**	**0.690**	0.214
**31**	-0.356	0.173
**32**	0.073	0.202
**33**	**0.846**	0.031
**34**	0.268	**0.526**
**35**	**0.786**	0.009
**Cronbach’s alpha**	**0.89**	**0.77**

Sum of items:	1.2.5.11.12.16.19.20.21.24.25.26.28.29.30.33.35	4. 10.15.17.22.23.34

## 4. Results

The Greek NDI-35 that was produced was administered to 200 nurses (144 women and 56 men). One hundred and eighty four nurses were TEI graduates and 64% had a more than 15 years professional experience (Table 3). The relative proportions of males and females reflect national patterns for the gender balance in nursing. The two factor analysis was indicated as a possible solution in the present study because of the discontinuity in the scree plot. These two factors explained 45.76% of variance. Values of the items in the two subscales were added and the sum was divided by the number of items incorporated in every factor. Two subscales arbitrarily called “clinical work” and “patient needs” emerged. In each subscale, the participants scored greater than the potential mean value with the mean “clinical work” subscale score being at 70.16 ±12.90 (a maximum of 85) and mean “patient needs” subscale at 21.49± 6.16 (Table 4). Considerable differences (*see italics in Table 4)* in scoring among different items were observed when the NDI-35 answers were compared to their Greek counterparts’ (Table 5). The final version of the GR-NDI-24 is present in the Appendix.

**Table 3:** Demographic features of nurses

	N	*%*
**Gender**		
Men	56	*28,0*
Women	144	*72,0*
Total	200	*100,0*
**Professional experience**		
0-5 years	18	*9,0*
5-10 years	30	*15,0*
10-15 years	24	*12,0*
15-20 years	38	*19,0*
>20 years	90	*45,0*
Total	200	*100,0*
**Educational level**		
University graduate	16	*8,0*
ΤECHNOLOGICAL INSTITUTIONS graduate	184	*92,0*
Total	200	*100,0*

**Table 4:** Mean values in the two subscales of NDI-35 (Greek version)

Subscales (range) N=200	Min.	Max.	Mean	Standard deviation
Clinical work (17-85)	22.00	85.00	70.16	12.90
Patient needs (7-35)	9.00	35.00	21.49	6.16

**Table 5:** Comparative presentation of mean scores/item in UK and Greece

Item	Mean (Greece)	Mean (UK)
1	3,46	1.09
2	4,11	1.05
3	2,52	3.61
4	3,06	3.51
5	3,99	1.16
*6*	*1,80*	*3.80*
*7*	*2,76*	*3.99*
8	3,34	2.46
9	3,28	3.27
10	2,86	1.82
11	4,28	1.48
12	4,38	1.06
*13*	*2,46*	*3.58*
14	1,88	4.04
15	2,78	1.65
16	3,91	1.31
17	3,16	1.13
18	3,37	3.27
19	4,07	1.68
20	4,13	1.07
21	4,32	1.58
22	3,33	1.68
23	2,60	2.12
24	3,99	1.35
25	3,97	1.33
26	4,16	1.12
27	3,45	2.15
28	4,32	1.06
29	4,44	1.13
30	4,26	1.47
*31*	*1,94*	*4.18*
32	3,04	1.38
33	4,63	1.21
34	3,86	2.05
35	4,21	1.09

## 5. Discussion

The factorial validity and internal reliability of the Greek version of NDI-35 is acceptable and permits the further study of its properties in a larger, representative and randomly selected sample in order to conceptualize Greek nurses’ attitudes and norms of caring. Despite its initial CDI-35 five factor design in the English version and the two factors finally added in the later and renamed version of NDI-35, the two subscales emerged through validation in its Greek version incorporate different items than the suggested ones. A two-factor structure for the NDI-35 was also clearly indicated for Spanish nurses and nursing students (Watson, 2003).

The discriminate factors “clinical work” and “patient needs” were obvious, instead of “important-unimportant” activities. Cultural variations may account for this discrepancy. Cultural variability could seriously affect a questionnaire design and the expected outcomes (Johnson, Cho, Holbrook, O’Rourke, Warnecke, & Chavez, 2006). Previous studies (Lea, Watson, & Deary, 1998) identified a psychosocial dimension with regard to the CDI-25 (item examples: ‘‘listening to the patient’’; ‘‘sitting with the patient’’), resembling the “patient needs” subscale in the present study. This underlying dimension perhaps represented a one-dimensional scale that was finally assessed in the present study.

A limitation of the present study is the fact that the participant groups were all selected on the basis of convenience; therefore, the extent to which the results may be generalized is limited. Moreover, the heterogeneity of patients may have had an influence which it is not easy to measure. One should keep in mind that a questionnaire can only be validated for a certain population, under certain conditions (Johnson, Cho, Holbrook, O’Rourke, Warnecke, & Chavez, 2006). Generalization of results should be treated cautiously, especially in the health sector, where great disparities and cultural differences exist between countries. Greek participants scored higher in most items, with exception of those items related rather to health system facilities than the nurse activity. “Keep in contact with a patient after discharge” and “Assure a terminally ill patient that he or she is not going to die” are some examples and reveal the necessity for supporting nursing care with a follow-up system and experienced supervisors. In the future, a more robust design should incorporate randomly selected samples from all health sectors as well as longitudinal measures of all participant groups. Based upon our results future studies can apply the translated version to Greek nurses and in different settings and construct a national norm for nursing care. This could lead to closer surveillance of health services quality and further changes in nursing schools curricula, for caring sciences to be developed as appropriate.
